# Understanding Mental Health, Vulnerabilities, and Coping in Older Koreans and Older Korean Americans

**DOI:** 10.1093/geroni/igaa057.2167

**Published:** 2020-12-16

**Authors:** Nan Sook Park, David Chiriboga, Barbara Yee

## Abstract

Although significant progress has been made in understanding mental health issues, racial/ethnic minorities are disadvantaged in terms of knowledge, attitude/stigma toward mental illness, and access to treatment. Older Koreans and Korean Americans are high-risk groups with great prevalence of stigma and limited access to mental services. The two groups share similarities as well as differences. For example, Older Korean Americans, a first-generation immigrant group, tend to share traditional values and beliefs with older Koreans. However, differences in social and cultural contexts, availability of social networks and resources, and access to health care systems present unique challenges and strategies. The purpose of this symposium is to enhance the understanding of critical issues in mental health among older Koreans and Korean Americans and identify challenges and strategies to promote mental health and well-being. Five studies conducted in Korea and the US will explore a variety of personal, social, and cultural factors related with mental health, based on quantitative and qualitative approaches. The diverse topics cover the mediating role of self-esteem in the relationship of stigma to emotional well-being, the effect of stress and coping on well-being, loneliness and negative family interactions, the relationship of life stressors and social capital on mental distress among older Korean Americans compared with other older Asian Americans, and community leaders’ attitude toward depression. The issues of vulnerabilities and resources will be discussed from the cultural perspectives as well as implications for future research and practice.

